# Patients’ perspectives of epilepsy care by specialists and generalists: qualitative evidence synthesis

**DOI:** 10.3399/BJGPO.2024.0072

**Published:** 2024-12-11

**Authors:** Charlotte L Cotterill, Andrew Booth, Jon M Dickson, Daniel Hind

**Affiliations:** 1 The University of Sheffield, Population Health, School of Medicine and Population Health, Sheffield, United Kingdom

**Keywords:** systematic reviews, epilepsy, patient perspectives, general practitioners, qualitative research

## Abstract

**Background:**

In the UK, epilepsy care involves both specialists (for example, neurologists) and generalists (for example, GPs). Policymakers typically consider that epilepsy care should be integrated and involve both specialists and generalists. However, few understand exactly how patients view and compare specialist and generalist care.

**Aim:**

To explore patient perspectives of specialist care and generalist care for epilepsy in a qualitative evidence synthesis.

**Design & setting:**

A systematic review of patient perspectives of epilepsy care. A qualitative evidence synthesis was conducted using an identified framework.

**Method:**

Systematic searches in five databases retrieved 17 eligible studies. Data were extracted and synthesised using framework analysis informed by the ‘United Model of Generalism’.

**Results:**

The following three themes were developed: ‘Epilepsy care can be burdensome’ (for example, through care fragmentation); ‘Patients' experiences of care is that care is not always accessible’ (for example, lack of a continuum between standardised and interpretive care); and ‘How care could change for people with epilepsy’ (for example, clinicians currently have insufficient time to deviate from protocol-driven care to address psychosocial needs). People with epilepsy frequently observe that generalists lack expertise in epilepsy management.

**Conclusion:**

This synthesis of patient experiences indicates recommendations should focus on improving communication and integration between specialists and generalists for epilepsy care. Patient experiences indicate specialist care risks being burdensome and generalist knowledge insufficient, requiring enhanced primary care clinician skills and improved awareness of patient psychosocial needs. The findings argue in favour of healthcare policies, materials, and tools to continually support patient perspectives in developing epilepsy services.

## How this fits in

Epilepsy care is more than just seizure control via anti-seizure medications, vagus nerve stimulation, or surgical intervention; broader patient needs should be addressed across the healthcare system, including primary care, secondary care, and tertiary care, considering the preferences of people with epilepsy themselves. This includes both specialist and generalist skills, which are considered here using a selected framework to synthesise the literature, with recommendations to clinicians on how this can inform patient-centred care in future. To our knowledge, patient perspectives of epilepsy care has never been systematically reviewed.

## Introduction

Availability of consultant neurologists varies considerably across the UK.^1^ Demand for specialist epilepsy care exceeds the availability of appointments, leading to long waiting lists, suboptimal care, and delays accessing imaging investigations.^2^ National audits have identified shortcomings in the care of people with epilepsy (PWE), with seizures accounting for 2–3% of attendances to urgent care and emergency departments, and many patients noted as taking older antiepileptic drugs.^3^

The National Institute for Health and Care Excellence (NICE) guidelines state that following the first seizure, adults should be urgently referred to *'a clinician with expertise in assessing first seizures and diagnosing epilepsy'.* ^4^ A neurologist with or without a subspecialty of epilepsy can make an epilepsy diagnosis,^5^ although owing to a number of differential diagnoses and epilepsy syndromes informing treatment and approach to managing seizure control, a subspeciality is beneficial.^6^ A study in the Wrexham area found that the rate of misdiagnosis was 5.6% for neurologists (which the study classified as specialist) and 18.9% by non-specialists.^5^

The World Health Organization (WHO) highlights generalists (primary care) as important for delivering whole-person and patient-centred care for PWE.^7^ Generalist input strengthens monitoring and evaluation of patients, which has been demonstrated to prevent epilepsy seizures following stroke.^8^ UK guidelines do not specify who delivers ongoing care to PWE, although it is typical for care to continue being delivered by specialists, some have commented on how care swiftly returns to primary care management,^5^ which is sometimes supported by epilepsy specialist nurses (ESN). Generalism is not synonymous with GPs (nor primary care),^9,10^ but primary care is considered the *'cornerstone of generalist practice*'.^11^ In the past, the Quality and Outcome Framework (QOF) has been used to incentivise greater primary care involvement in long-term conditions, including epilepsy.^12^ Following removal of most epilepsy QOF indicators in 2014, annual primary care reviews for epilepsy have fallen significantly from 95% in 2010 to 14% in 2016^13^ and the role of primary care for PWE has become diminished.

Within a whole-system approach to care, specialist care and generalist care have been described as overlapping vertical (specialist) and horizontal (generalist) planes, working together to support clinicians.^14^ The ‘United Model of Generalism’,^15^ describes a similar structure that includes specialist and multidisciplinary involvement, describing the vertical and horizontal planes as clinical decision making and organisation of care. The inclusion of both specialist and generalist knowledge, plus organisation and delivery of care, makes the United Model of Generalism^15^ highly relevant to use as an *a priori* conceptual framework^10^ for considering PWE’s experiences of care.

Understanding patient views on the respective roles of specialists and generalists is critical for designing epilepsy services that are responsive to patient needs and preferences, supporting more integrated patient-centred care.^16^ Since 2014, publications of patient perspectives have increased.^17^ Patient-centred care has been criticised for often assuming patient perspectives through the actions of health professionals and their decision making, rather than directly through consideration of patient experiences.^16^ However, the patient perspective remains under-represented in assessments of epilepsy care models, despite the relevance of this data to health system goals about care coordination and patient centredness for chronic conditions. Similar research of other complex conditions requiring neurological care, such as multiple sclerosis, has been considering the preferences of patients for more than 20 years and is still ongoing.^18,19^

Using a qualitative evidence synthesis (QES), we aimed to answer two related questions. First, how do patients view generalist versus specialist models of epilepsy care? Second, what do patients consider the strengths and weaknesses of specialist and generalist epilepsy care? Our objectives were as follows: (1) to systematically search a broad range of literature surrounding patient perspectives of epilepsy care organisation and management; (2) to use full-text screening techniques to identify eligible articles comparing specialists with generalists from accounts of PWE; and (3) to conduct a QES of eligible studies to achieve a consensus as to what generalist and specialist care provides, and which is preferred by patients.

## Method

A QES^20^ was conducted using the United Model of Generalism^15^ as an *a priori* framework ^21^ to extract data from the included studies. Reeve and Byng’s model consists of nine interrelated declarative statements^15^ (*a priori* propositions). These were a starting framework for coding data from the literature.

### Eligibility criteria

The inclusion and exclusion criteria for study selection are listed in Table 1. Patients’ views of generalist and specialist epilepsy care is a specific topic, unlikely to be the primary focus of a primary qualitative study. The views collected from patients in the included studies are considering epilepsy care delivered by doctors, in epilepsy specialist settings (for example, neurologists with and without a subspeciality in epilepsy, or doctors in generalist settings (for example, a GP but also non-epilepsy specialists). The decision to exclude nurses and epilepsy specialist nurses is because they represent a multifaceted role often acting as an intermediary between specialism and generalism, which is difficult to distinguish in patient perspectives. For retrieval, a study needed to report the perspectives of patients in relation to care of their epilepsy but this did not need to be its focus. Full-text of all the articles meeting this were then examined to establish the presence (or not) of data on specialist or generalist care.

**Table 1.** Eligibility criteria

Eligibility criteria	Include	Exclude
Only qualitative studies to be included in the review	Studies with recognised methods of qualitative data collection and analysis	Quantitative data collection methods
	Mixed-method studies containing recognisable qualitative data	Mixed-method studies without recognisable qualitative data
Eligible studies must include people living in the UK with epilepsy, sharing their views and perceptions of the care they receive	Participants of included studies must be adults (aged >18 years) with an epilepsy diagnosis in the UK	Participants who are adults experiencing seizures but no epilepsy diagnosis
	Perspectives of people with epilepsy on their own care, and the insights to the organisation of care	Perspectives from carers of people with epilepsy
The patients included in the study must be sharing views related to their experiences with doctors (in either specialist or generalist settings). Views may either be explicitly related to specialist or generalist care, or include insights that can be interpreted in the context of specialist or generalist models of care	Perceptions and views delivered by a generalist doctor (for example, GP) or specialist doctor (for example, neurology consultant)	Perceptions and views delivered by nurses and allied healthcare professionals
	Views of patients when the review team agrees that care delivered by (generalist) doctors is implicitly included	

### Search strategy

Database searches of PubMed, MEDLINE, PsycInfo (Ovid), Scopus, Web of Science, and Embase (Ovid) were undertaken in December 2022. References for all included articles were checked for further relevant studies (backward citation tracking). The searches were compiled in three separate stages, for the three eligibility criteria, which were combined into the search strategy components (see Table 2 for PubMed search strategy). The topic of the search was epilepsy; participants and setting were PWE in the UK; and study design and phenomenon searched for qualitative studies that included patient, as opposed to healthcare provider, perspectives.

**Table 2.** Search strategy

Databases	Search strategy component	Search terms
PubMed	Topic	Epilepsy [MeSH] OR Seizures [MeSH] OR Epilep*[Text Word] OR seizure*[Text Word] OR Aura*[Text Word]
	Participants and setting	Great Britain[MeSH] OR "national health service"[Title/Abstract] or “nhs”[Title/Abstract] OR english[Title/Abstract] not (published or publication* or translat* or written or language* or speak* or literature or citation*) OR (gb[Title/Abstract] OR "g.b."[Title/Abstract] OR britain*[Title/Abstract] OR (british*[Title/Abstract] NOT "british columbia"[Title/Abstract]) OR uk[Title/Abstract] OR "u.k."[Title/Abstract] OR united kingdom*[Title/Abstract] OR (england*[Title/Abstract] NOT "new england"[Title/Abstract]) OR “northern ireland*”[Title/Abstract] OR northern irish*[Title/Abstract] OR scotland*[Title/Abstract] OR scottish*[Title/Abstract] OR ((wales[Title/Abstract] OR "south wales"[Title/Abstract]) NOT "new south wales"[Title/Abstract] OR "new south wales"[Title/Abstract]) welsh*) OR uk[AD] OR "united kingdom"[AD] OR (England[AD] NOT “New England”[AD]) OR (Wales[AD] NOT “New South Wales”) OR Scotland[AD] OR “great britain”[AD] OR “Northern Ireland”[AD] AND
	Study design/phenomenon	(((“semi-structured” or semistructured or unstructured or informal or “in-depth” or indepth or “face-to-face” or structured or guide) OR (interview* or discussion* or questionnaire*))).ti,ab. or (focus group* or qualitative or ethnograph* or fieldwork or “field work” or “key informant”).ti,ab. or interviews as topic[MeSH] / or focus groups [MeSH] or narration[MeSH] or qualitative research[MeSH] OR Attitude* OR experience* OR perception* OR view* OR Patient Acceptance of Health Care[MeSH] OR ((patient* or consumer*)).ti,ab.OR Consumer Advocacy [MeSH] OR consumer satisfaction[MeSH] OR Consumer Satisfaction OR attitude to health[MeSH] OR Consumer Participation/ OR Patients/px OR professional-patient relations[MeSH] OR health knowledge, attitudes, practice[MeSH] OR patient advocacy[MeSH] OR patient education as topic[MeSH] OR Patient Preference[MeSH] OR patient-centred care OR self care OR self concept [MeSH] OR self-help groups[MeSH] OR “patient education” OR “patient satisfaction”

### Study selection

A subsection of retrieved studies were screened by title and abstract independently by two authors, then compared and discussed until they reached an agreement. The remainder were divided and screened by title and abstract individually by the same two authors. Articles found to be eligible, based on all inclusion criteria, or if the study abstract did not include enough information were retained for full text screening.

Full-text screening followed the same eligibility criteria and process that the authors followed for screening by title and abstract. A consensus meeting on eligibility for the final included studies took place before data extraction. At this stage, the final sample of selected studies were graded according to relevance with a data richness scale used for purposeful sampling in QES.^22,23^ Studies were graded for richness from 1–5 based on relevance to the *a priori* propositions, 1 representing very limited data, and 5 being a large amount of applicable, in-depth, qualitative data.^22^

### Synthesis

Qualitative data for analysis were extracted in substantive extracts rather than line-by-line coding. Relevant data were in the form of verbatim extracts from study participants, author’s observations of the study participant’s responses, and author’s observations with supporting verbatim extracts. Data were added to a data extraction form (see supplementary Appendices) with the *a priori* propositions used as a framework for synthesis. Data that did not fit the *a priori* propositions from the theoretical model, but were still applicable to the review question were still included. These were synthesised into review themes, making new propositions if needed until all data could be accounted for.^23^

### Reflexivity

The work is complementary to a funded studentship on epilepsy in primary care (CLC), and an author is an academic GP (JMD) working in and researching epilepsy in primary care. This review made efforts to minimise bias by using a pre-existing theoretical framework^15^ that explicitly outlines the importance and integration of specialist knowledge in varied settings, so as to objectively analyse the review data.

### Critical appraisal

A critical appraisal of all included articles was independently undertaken, using the Critical Appraisal Skills Programme (CASP)^24^ qualitative checklist to assess study validity, rigour, and usefulness of findings. Key domains appraised included aims and appropriateness of qualitative methodology, research design, recruitment strategy, data collection, researcher reflexivity, ethical considerations, data analysis, findings, and value of the research. Studies were not excluded based on the appraisal, but appraisal findings helped guide synthesis.

## Results

**Figure 1.**
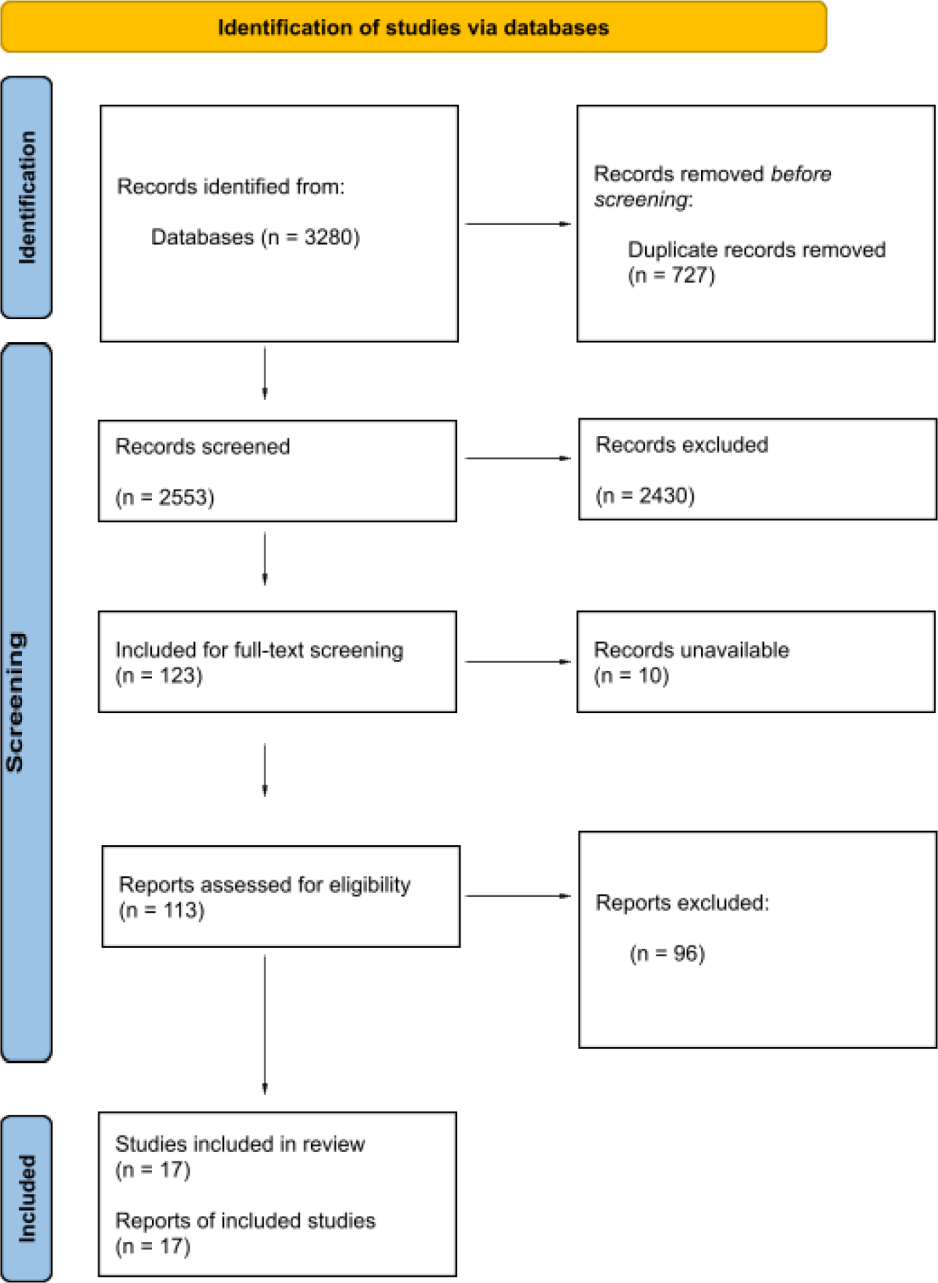
PRISMA flow diagram From: Page MJ, McKenzie JE, Bossuyt PM, et al. The PRISMA 2020 statement: an updated guideline for reporting systematic reviews. BMJ 2021;372:n71. doi: 10.1136/bmj.n71.

The Preferred Reporting Items for Systematic Reviews and Meta-Analyses (PRISMA) flow diagram (Figure 1) presents the process of database searching and screening of articles. The database searches retrieved 3280 references, 727 were duplicates. The remaining 2553 were screened by a reviewer to establish they were definitely qualitative, with 2171 removed. The remaining 382, including an overlap of 26%, were divided between the two reviewers who screened by title and abstract, with 123 included for full-text screening. Ten studies were unavailable as electronic full text, 113 were examined for relevance to the review objectives, qualitative data collection methods, analysis, and data.^23^ The final sample was 17 studies (Supplementary Table S1).

### Synthesis of findings

Table 3 outlines the *a priori* propositions from the United Model of Generalism^15^ and how they were used to analyse primary data. Table 4 provides an overview of how the review findings in Table 3 are organised under the review themes.

**Table 3.** Qualitative evidence synthesis proposition table, sub-propositions, and supporting studies

A priori declarative statements (The United Model of Generalism)	Statement of review findings and supporting studies
*'Standardised, disease focused* [guideline] *medical care, even done well can have a burdensome effect on individuals'.* ^15^ p. 292	Patients find their experiences of specialist care burdensome^25,27–30,37,39,49^
	People with epilepsy find fragmentation between specialists and generalists problematic^31–33^
*'Burden can be over-investigation, overtreatment, or a failure to address illness experiences*.'^15^) p.292	Patients support the definition of burden as over-information, overtesting and failure to address illness experiences of people with epilepsy^29–31,34–39,41^
*'A continuum from single problem, accessible care through to integrated care bringing together different skills and teams.'* ^15^ p.292	A continuum of standardised accessible and integrated care was not evidenced from patient perspectives of epilepsy care^25,27,28,31,34,37,41^
*'A continuum from standardised replicable, and often evidence informed, to highly interpretive care.'* ^15^ p.292	People with epilepsy feel replicable care beyond medication prescribing is difficult to achieve^32,34,41^
*'Primary care clinicians are often required to adjust their manner to respond to a particular patient’s needs.'* ^15^ p.292	Clinicians do not have time to deviate from standardised care^25,37,41^
*'Single problem/standardised care delivers low intensity accessible care at volume, mainly but not only in primary care.'* ^15^ p.292	Inconclusive views on how satisfactory patients find generalist epilepsy care^34,36,37,41^
*'Some patients need ready access to professionals skilled in interpretive practice.'* ^15^ p.292	–
*'*[patients] *may benefit from signposting to* ***services outside of medical care*** *, but generally don’t need high levels of integrated care across medical teams.'* ^15^ p.292	Clinicians do not adjust their approach to consider people with epilepsy’s psycho-social care^27,31,32,34^
–	People with epilepsy feel critical of generalists who lack awareness of epilepsy^32,33,39^
'*Patients with chronic complex care needs, especially those with a diminished capacity to manage daily living need both integrated and interpretive care.*'^15^ p.292	–

**Table 4.** Overview of review themes

Theme	Sub-theme (propositional finding)	Synopsis
Epilepsy care can be burdensome	Patients find their experiences of specialist care burdensome	Standardised care pathways are burdensome to people with epilepsy as they are overly focused on specialist care
	People with epilepsy find fragmentation between specialists and generalists problematic	Poor communication between specialists and generalists result in greater burden for people with epilepsy
	Patients support the definition of burden as over-information, overtesting, and failure to address illness experiences of people with epilepsy	Burden continues to be multifaceted, and occurs in both specialist and generalist care
Patients' experiences of care is that care is not always accessible	A continuum of standardised accessible and integrated care was not evidenced in patient perspectives of epilepsy care	There was no real life evidence for how care can be both accessible and integrated. Findings discussed continue to indicate that specialist and generalist care are experienced differently, with flaws noted when each works without input from the other
	People with epilepsy feel replicable care beyond medication prescribing is difficult to achieve	Replicable care is key to the United Model of Generalism, but was not found to be consistent between specialist and generalists
How care could change for people with epilepsy	Clinicians do not have time to deviate from standardised care	The approach of clinicians is often limited by availability of time to deviate from standard care plans. This presents itself in different ways for specialists and generalists
	Inconclusive views on how satisfactory patients find generalist epilepsy care in addressing uncertainty	Broader patient needs are important to consider for people with epilepsy’s mental wellbeing and lifestyle, but are often overlooked by both generalists and specialists
	Clinicians do not adjust their approach to consider people with epilepsy’s psychosocial care	People with epilepsy continue to advocate that greater awareness of epilepsy is needed across all healthcare settings
	People with epilepsy feel critical of generalists who lack awareness of epilepsy	Continuing need for a conclusive view on generalist care given the broad perceptions of generalist care identified from included studies

### Epilepsy care can be burdensome

#### Patients find their experiences of specialist care burdensome

PWE reported burden during standardised care for diagnosis and treatment. The referral process itself was difficult, delaying diagnosis.^25^ Neurologists often fail to adequately explain outcomes, further burdening patients.^26–28^ When patients experience burden from standardised care, this drives some PWE to rely on *'distressing'* ^29^ alternative care from emergency services.^29,30^

#### PWE find fragmentation between specialists and generalists problematic

Beyond the *a priori* proposition, patients’ experiences of fragmented care add to their burden. Fragmentation between specialists and generalists is problematic, the multifaceted nature of epilepsy^31^ requires changes to be shared between providers, increasing patients’ *'hard work*'^32^:

*'The need for information increased when informants were faced with life-changing events such as puberty, pregnancy or menopause. Those diagnosed at a young age or whose parents could not communicate in English were further disadvantaged.'* ^33^

#### Patients support the *a priori* definition of burden as over-information, overtesting, and failure to address illness experiences of people with epilepsy

Over-information was associated with specialist care for newly diagnosed patients^34^ and those with established care routines.^35^ *'When I went to the epilepsy clinic, that made me ten times more aware and ten times more scared as well … ’ (Johnny, 20).* ^30^ Overtreatment, particularly in generalist care, occurs where patients have difficulty seeing the same doctor each visit,^34^ compounded by communicating issues with medication.^31^ Patients highlight a failure of clinicians in both specialist and generalist settings to address their illness experiences.^29,36^ *'They try you on tablets but they don’t listen to how they make you feel.'* ^37^ PWE need support with coping strategies and overly restrictive advice.^37,38^

### Patients' experience is that care is not always accessible

#### A continuum of standardised accessible and integrated care^15^was not evidenced from patient perspectives of epilepsy care

Integrated care^15^ was unusual to come across in epilepsy care^39^; perceptions of care were polarised as either specialist or generalist.^38,40^ Patients found it challenging to access specialist knowledge; for example, *'there are nowhere near the number of neurologists or epileptologists in the country that are needed'.* ^27^ Some PWE felt that reliance on specialist decision making creates opportunities for mistakes.^31^

#### PWE feel replicable care beyond medication prescribing is difficult to achieve

According to PWE, advice given by generalists differs from that given by specialists.^41^ Consistent care recommendations are important if PWE are to reconcile medications with other needs.^32^ Replicable care from specialists was thought to involve medication only, without wider aspects of care.^34^

### How care could change for PWE

#### Clinicians do not have time to deviate from standardised care

PWE perceive specialists as being unaware of other epilepsy services and not allowing time to discuss them.^26,41^ GPs often do not take the time to change their approach to patient needs. Consultations are a one-way medical process, without adjusting to give broader advice.^41^

#### Clinicians do not adjust their approach to consider PWE’s psycho-social care

Generalists and specialists do not modify their approach according to a patient’s lifestyle^34^ and mental wellbeing:^31^

*'A lot of my fits are caused by depression, which* [is] *caused by the amount of drugs I am taking … They just don’t really look at the whole picture.'* ^41^

Whole-person care, which seeks to address broader patient needs, should thus consider additional needs, such as contraception and conception choices.^27,32^

#### PWE feel critical of a lack of awareness

A lack of epilepsy knowledge, beyond epilepsy specialists, limits all aspects of patient care, not just epilepsy management.^33^ Patients criticised generalists’ poor epilepsy awareness, *'They know nothing as far as I'm concerned!',* ^37^ but praised awareness of broader issues outside of their epilepsy care, such as social aspects of care and accessing free prescriptions^37^. During labour, PWE were vulnerable to serious complications as maternity specialists did not consider seizures.^32^

#### Inconclusive views on how satisfactory patients find generalist epilepsy care in addressing uncertainty

Annual check-ups, prescribing, and other generalist care was considered easily accessible.^34,37,41^ '*My GP is excellent really. He’s not an expert on everything because they can’t be.'* ^39^ Yet doubts persist as to whether generalists satisfactorily address uncertainties. PWE welcomed further access to advanced epilepsy knowledge when faced with ambiguous symptoms.^34^ PWE identified insufficient exploration of epilepsy triggers by generalists.^37,41^

## Discussion

### Summary

This QES found three themes on PWE’s perspectives of care. Each theme includes sub-themes, which were developed from both the review literature and *a priori* framework. The results indicate the importance of improving communication between specialists and generalists as well standardised care pathways influencing patient experiences.

The review found care burdens PWE, failing to address their needs. In particular, PWE find fragmentation between specialists and generalists problematic. This finding has implications for service design, professional education, clinical communication, and health policy aimed at improving care integration, continuity, and patient–clinician relations. Fragmentation between specialists and generalists aligns with findings from other clinical areas. For example, in stroke care, despite working in overlapping roles, specialists and generalists often remain siloed without adequate knowledge of the patient’s additional needs.^42^ Fragmentation points to a need for improved communication and role clarity between providers to support continuity and coordination from a patient perspective.^16^

### Strengths and limitations

This QES is the first to summarise patient perspectives on specialist and generalist models of epilepsy care. It uniquely applies the United Model of Generalism,^15^ to extract and synthesise relevant data capturing PWE’s experiences, allowing novel interpretive insights into care fragmentation, accessibility, and quality alongside inductively derived themes on improving epilepsy services. The data richness grading^22^ was useful when sampling broadly from articles which have relevant data but with research objectives different to the review. Rigour was established through systematic search methods, critical appraisal of all included studies, and duplicated stages of data analysis between authors. The large, diverse sample of patient perspectives strengthens transferability.

In this review, we included studies regardless of epilepsy subtype or seizure type. Although epilepsy is a highly heterogeneous disorder and different subtypes create different challenges for patients and for clinicians, and they have different effects on quality of life, all forms of epilepsy are characterised by recurrent seizures; this is the core feature of all the epilepsies. We think that our approach was justified by the themes we identified in the review, which apply equally well to all epilepsy subtypes.

An issue not addressed in this review is whether using specialist and generalist as labels for those with and without expertise in epilepsy adds to polarisation^11^ between clinicians. The terms specialist and generalist, especially specialist, are consistent with the language used by patients themselves in patient representative authored articles^43^ and thus suitable in this context. However, the authors acknowledge limitations on using language such as specialist and generalist.

It is important to acknowledge that our findings reflect patient perceptions of their care experiences, rather than objective evidence of specialist and generalist epilepsy services. Amid increasing recognition of patient experience as a key quality outcome, it continues to be underreported and poorly understood.^16^ Patient views offer valuable insights for informing service design and policy; they may not always align with provider perspectives or capture the full complexity of health system issues. Future research should triangulate patient experiences with other measures to more comprehensively evaluate the real-world impacts of different care models.

We acknowledge that our findings may not fully reflect the diversity of patients given the heterogeneity in epilepsy types, severity, and care needs. PWE are a diverse group and factors such as location, socioeconomic status, comorbidities, and access to services can significantly shape patient journeys and perceptions. Future research should explore in more depth how these contextual factors intersect with patient views on generalist and specialist care, including access to and awareness of epilepsy organisations who support service provision outside of medical care.

### Comparison with existing literature

Previous surveys have repeatedly highlighted further information needs among PWE.^44,45^ Gaps and missed information at an organisational level can in part be attributed to poor multidisciplinary care. This was found to be common and when continued leads to risks for patients: an included study indicated that failure to provide adequate information led to late action in changing harmful epilepsy medication during pregnancy.^32^

### Implications for research and practice

There were very few qualitative studies in which the primary objective of the research was to gather and understand the perceptions of patients on their epilepsy care. Patient-reported outcomes and experience measures (PROMs and PREMs) are criticised for epilepsy as being poorly developed.^46^ This indicates a greater need for future qualitative research on patient perspectives of care, particularly as the NHS Long Term Plan continues to be put in place. Further research is recommended with a greater focus on gaps between specialist and generalist care, including whole-person care with consideration for PWE’s lifestyles.

Based on the findings of this review, support should focus on educating clinicians and improving awareness of epilepsy, which is a current barrier to delivering good quality epilepsy care. Patient-centred care is an important value of the NHS Constitution for England, which this review indicates is currently not being met sufficiently. Thus several concrete recommendations emerge from the findings. The NHS Long Term Plan^43^ aims to improve out-of-hospital care, supporting primary medical and community health services. These are all areas to prioritise for epilepsy care, which forthcoming revisions of the NHS Rightcare Epilepsy Toolkit^47^ should consider based on the review findings regarding fragmentation, inaccessible specialist care, and limited generalist knowledge.

Evidence suggests GPs with extended roles (GPwER) working collaboratively with specialists and epilepsy specialist nurses (ESNs) could provide an optimal intermediary model between primary and secondary epilepsy care.^48^ As medical care becomes increasingly modularised and personalised, generalist intermediaries will be essential for integrating diverse information sources, arranging multidisciplinary care and supporting patient decision making.^46^ Integrated Care Boards (ICBs) should consider funding partnerships between GPwER and ESN to enhance the primary care and secondary care interface.

This synthesis underscores the urgent need to address persistent fragmentation, accessibility barriers, and limited patient-centredness in current epilepsy care models. The patient perspective powerfully illustrates the real-world consequences of these system-level issues. To meaningfully improve patient experiences and outcomes, future policies and services must prioritise seamless coordination between specialists and generalists, enhanced access to specialist expertise at the primary care level, proactive identification of patient psychosocial needs, and ongoing solicitation of patient input. GPs with specialist roles in epilepsy (now referred to by the Royal College of General Practitioners [RCGP] as 'GPwER') work collaboratively with epilepsy specialists and ESNs, offering a promising solution to bridge existing gaps.^48^ With focused reforms guided by patient voices, we have an unprecedented opportunity to realise the promise of integrated, person-centred epilepsy care.

## Trial registration number

The review protocol was registered with PROSPERO (CRD42022382047).

## Data

N/A
